# Production, Characterization and Applications for *Toxoplasma gondii*-Specific Polyclonal Chicken Egg Yolk Immunoglobulins

**DOI:** 10.1371/journal.pone.0040391

**Published:** 2012-07-12

**Authors:** Álvaro Ferreira Júnior, Fernanda M. Santiago, Murilo V. Silva, Flávia B. Ferreira, Arlindo G. Macêdo Júnior, Caroline M. Mota, Matheus S. Faria, Hercílio H. Silva Filho, Deise A. O. Silva, Jair P. Cunha-Júnior, José R. Mineo, Tiago W. P. Mineo

**Affiliations:** Laboratory of Immunoparasitology, Institute of Biomedical Sciences, Universidade Federal de Uberlândia, Minas Gerais, Brazil; Department of Medical Microbiology and Immunology, United States of America

## Abstract

**Background:**

*Toxoplasma gondii* may cause abortions, ocular and neurological disorders in warm-blood hosts. Immunized mammals are a wide source of hyperimmune sera used in different approaches, including diagnosis and the study of host-parasite interactions. Unfortunately, mammalian antibodies present limitations for its production, such as the necessity for animal bleeding, low yield, interference with rheumatoid factor, complement activation and affinity to Fc mammalian receptors. IgY antibodies avoid those limitations; therefore they could be an alternative to be applied in *T. gondii* model.

**Methodology/Principal Findings:**

In this study we immunized hens with soluble tachyzoite antigens of *T. gondii* (STAg) and purified egg yolk antibodies (IgY) by an inexpensive and simple method, with high yield and purity degree. IgY anti-STAg antibodies presented high avidity and were able to recognize a broad range of parasite antigens, although some marked differences were observed in reactivity profile between antibodies produced in immunized hens and mice. Interestingly, IgY antibodies against *Neospora caninum* and *Eimeria* spp. did not react to STAg. We also show that IgY antibodies were suitable to detect *T. gondii* forms in paraffin-embedded sections and culture cell monolayers.

**Conclusions/Significance:**

Due to its cost-effectiveness, high production yield and varied range of possible applications, polyclonal IgY antibodies are useful tools for studies involving *T. gondii.*

## Introduction

*Toxoplasma gondii* is a well-adapted parasite of warm-blooded hosts, including human and production animals. The parasite has distinct developmental stages, such as the fast-replicating tachyzoites that are present in the acute phase of infection, while the slow-replicating bradyzoites form tissue cysts in muscular and nervous tissues during the chronic phase of infection [1]–[2]. Infection usually takes place after accidental ingestion of raw or undercooked meat containing tissue cysts, oocyst-contaminated food or water, and by transplacental passage of tachyzoites in active infection during the gestational period [3]–[4]. Congenital infection may lead to neurological disorders, ocular disease and/or fetal death [5]. *T. gondii* is also a major cause of infectious abortion and induces clinical disease in production animals, especially goat, sheep and swine, elevating production costs and resulting in considerable impact on the livestock industry as well as the public health [6].

Immunized hens transfer circulating antibodies from blood to egg yolk by specific receptors on the surface of the ovarian follicle [7]. Immunized hens may continue to produce antibody-rich eggs for up to two years - also known as IgY, which shares great similarity to mammalian IgG, and is a reasonably stable bivalent protein with estimated molecular weight around 180 kDa [7]–[8]. Egg yolk antibodies have been shown to be effective in immunohistochemical assays and for recognition of specific epitopes [9]. Polyclonal IgY have been previously described for the purification of polyepitope vaccine candidates against *Plasmodium falciparum* [10], passive immunization against *Eimeria acervulina* [11], immunoprophylactic protocols against influenza virus [12], and analysis of tumoral biomarkers [13], among others.

Specific polyclonal antibodies generated in mammals against *T. gondii* have been extremely useful in the last decades to unravel several aspects related to the parasite biology as host-parasite interactions, parasite invasion and replication mechanisms, candidate epitopes for diagnosis, characterization of *T. gondii* virulence factors and candidate protein for immunoprophylactic assays [14]–[17].

Here, we hypothesized whether polyclonal IgY could be a useful tool in the *T. gondii* model. In that sense, we produced, characterized and standardized distinct protocols for IgY application, using polyclonal IgY antibodies raised against *T. gondii* soluble tachyzoite antigen (STAg).

## Results

### Purification of Polyclonal IgY Antibodies

**Figure 1:**
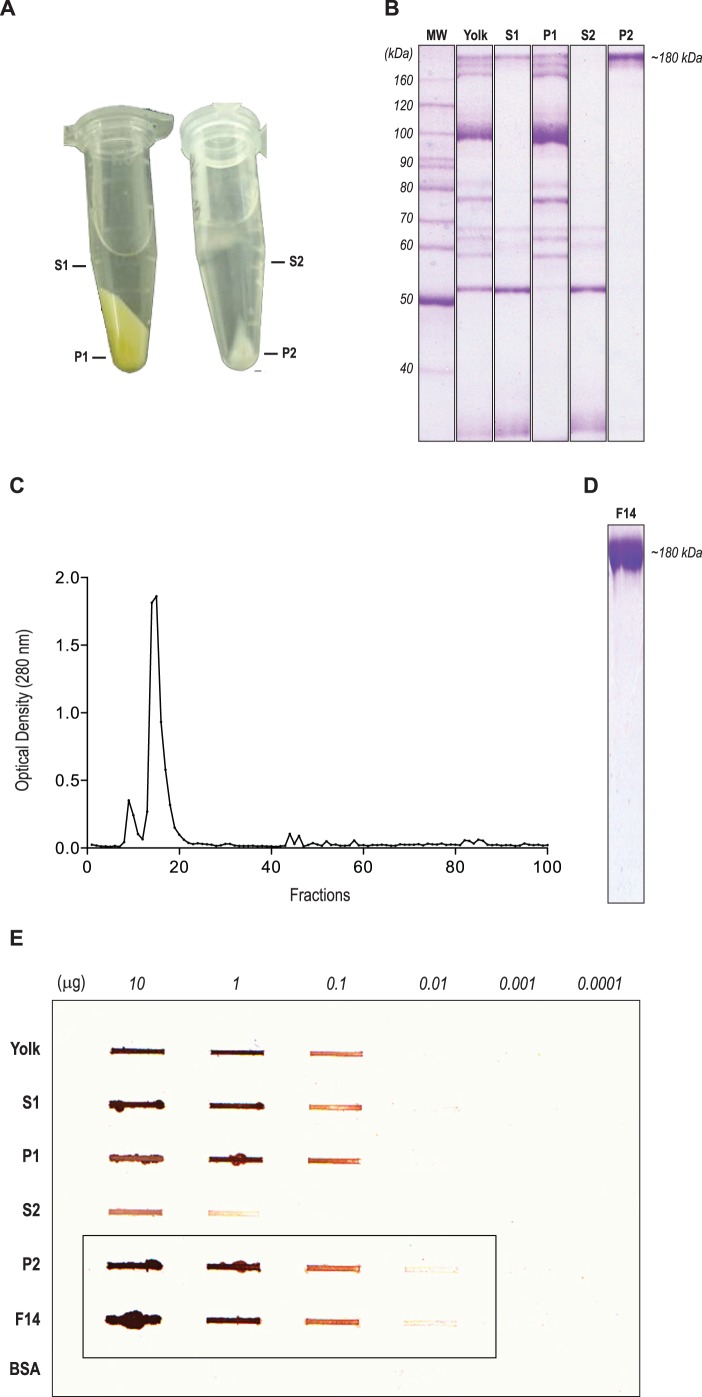
Purification of egg yolk antibodies. (**A**) Separation of the water soluble fraction (S1) from a lipid-rich precipitate (P1), after the incubation of crude egg yolk with acid water (pH 5.0–5.2); S1 precipitation by salting-out (19% Na_2_SO_4_) produced an enriched IgY pellet (P2) and a supernatant with contaminants (S2). (**B**): Purity degree of IgY samples determined by SDS-PAGE (8%), demonstrating that salting-out protocol produced high purity IgY antibodies. (**C**) Size-exclusion chromatography of the P2 fraction, with peak of IgY between 13th and 19th fractions, where (**D**) the 14th fraction presented the highest degree of purity. (**E**): IgY enrichment conferred by Slot-blot assay, IgY was detected until the concentration of 0.01 µg of protein (box). Bovine sera albumin (BSA) was used as negative control.

The first step consisted of the purification of IgY antibodies from egg yolks in order to obtain the highest yield and purity. Antibodies were separated from other constituents of the egg yolk by the addition of acid water and later precipitated by salting-out (19% sodium sulfate) (Fig. 1A). Although enriched in IgY antibodies, the water soluble fraction (S1) still presented undesirable low molecular weight proteins after acid water precipitation of pooled egg yolk (Fig. 1B, lane S1) that were eliminated after salting-out step (Fig. 1B, lane P2), presenting an average protein concentration of 4 mg/mL. We recovered 115 eggs from four STAg-immunized chickens during a 49-day interval. Each egg presented an average of 12 mL of yolk and the mean yield was 48 mg of polyclonal antibody per egg. Precipitated antibodies contained in P2 fraction were further submitted to purification by exclusion-size chromatography (Fig. 1C), resulting in a protein peak between the 13th and 19th fractions, although elevated purity was detected mainly in the 14th fraction (F14), as it may be observed in its SDS-PAGE profile (Fig. 1D). Thus, the used protocol was able to retrieve a mean of 4 mg of total IgY from 1 ml of crude egg yolk. Slot-blot assays confirmed IgY in fractions from salting-out and gel filtration purified fractions until the concentration of 110 ng of protein (Fig. 1E).

### Kinetics and Avidity of IgY Anti-STAg Antibodies

**Figure 2:**
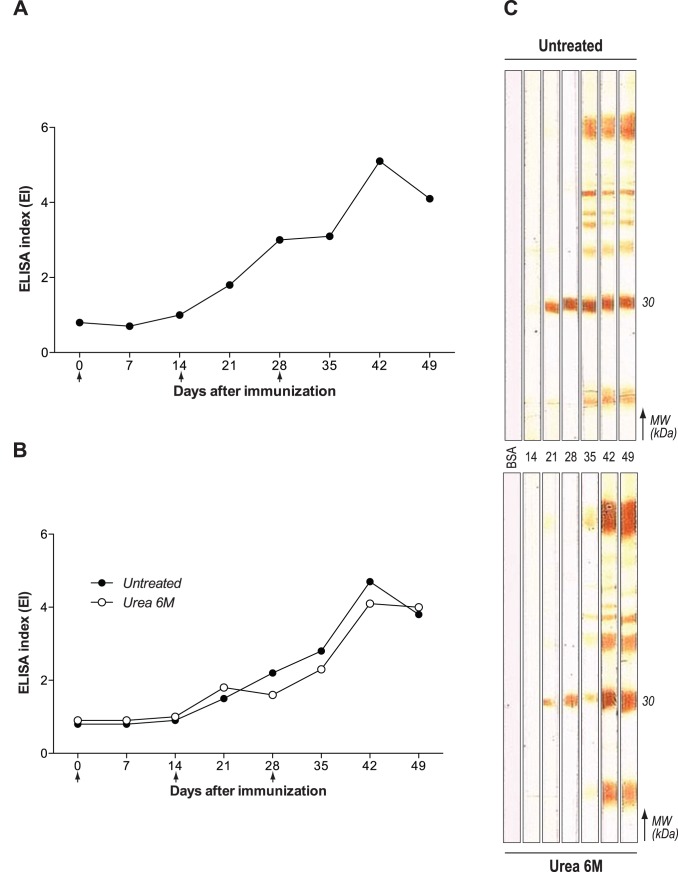
Production kinetics and avidity of IgY anti-STAg antibodies. Indirect ELISA showing (**A**) kinetics of chicken IgY and (**B**) IgY avidity maturation with 6M urea-treated (•) or untreated (○) immunocomplex. Black arrows indicate the three immunization doses. Samples with ELISA Index (EI) equal or superior to 1.2 were considered positive. (**C**) 1D-immunoblot presenting recognition kinetics (from 14 to 49 days p.i.) of STAg proteins by IgY and its respective avidity maturation, evaluated by 6M urea treatment of immunocomplexes.

After purification of an enriched IgY fraction, kinetics and avidity of the purified antibodies were evaluated by indirect ELISA. We verified that immunized hens presented detectable levels of specific IgY starting on day 21 after the first immunization (p.i.). The production of IgY anti-STAg increased significantly along the next weeks, and developed a progressive and sustained response, reaching its peak on day 42 p.i. (Fig. 2A). High avidity of IgY anti-STAg antibodies was already observed in the first reactive samples, at 21 days p.i., as demonstrated by the lack of significant differences in EI values between 6 M urea-treated and untreated samples (Fig. 2B). The antigen recognition repertoire and avidity maturation of IgY anti-STAg were also verified by immunoblot. Similar to ELISA, reactivity was first observed on day 21 after immunization, with strong staining of a 30 kDa protein (Fig. 2C). From the 35^th^ day onwards, IgY anti-STAg antibodies recognized a broad range of *T. gondii* antigens. The 6 M urea treatment presented a reduction in staining intensity of the described antigens between the 21^st^ and 35^th^ days p.i., with normal reactivity being observed after that period (Fig. 2C).

Mice immunized by the same route present a similar antibody seroconversion kinetics, with reactivity to STAg being detected on 14^th^ day p.i., preceded by initial p30 recognition on 7^th^ day p.i. in serum samples (Fig. S1).

### Applications for IgY anti-STAg Antibodies

**Figure 3:**
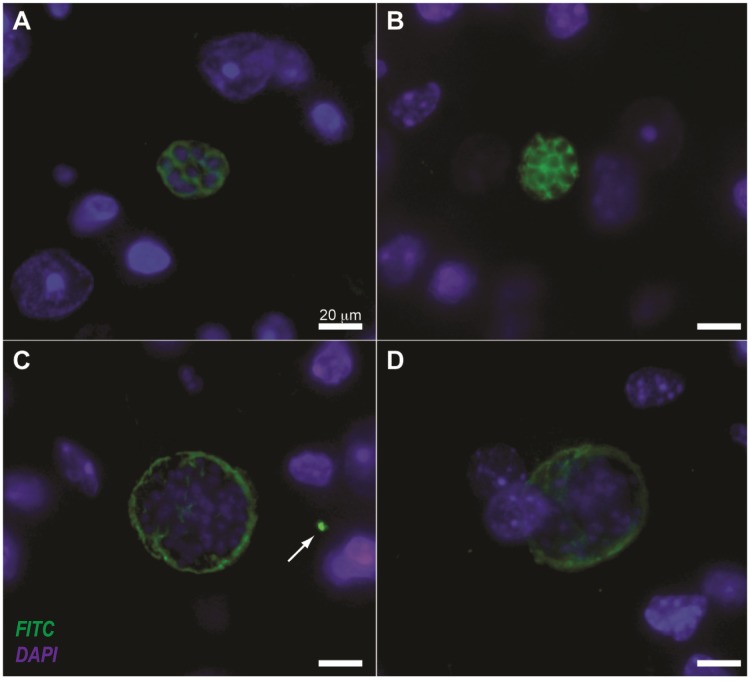
Immunohistochemistry for *T. gondii* detection using polyclonal IgY anti-STAg antibodies. Paraffin-embedded brain sections of mice chronically infected with ME-49 strain were incubated with IgY anti-STAg and rabbit IgG anti-IgY conjugated to fluorescein isothiocyanate (FITC). (**A** and **B**), segmented parasitophorus vacuoles were detected into host cell cytoplasm around the DAPI-stained nucleus (blue). (**C** and **D**), IgY anti-STAg antibodies strongly recognized antigens on outer walls of tissue cysts and free tachyzoites (**C**, arrow).

Once we were able to obtain pure, high avidity IgY anti-STAg antibodies, we aimed to verify potential applications of those antibodies in *T. gondii* model. The standardized immunohistochemical assay using IgY anti-STAg antibodies was able to detect antigenic distinct structures in brain tissue sections, as follows: (i) parasitophorous vacuoles, demonstrated as rounded and compartmentalized structures (Figs. 3A and 3B); (ii) tissue cysts, represented by a wall bright fluorescence (Figs. 3C and 3D); and (iii) free tachyzoites (Fig. 3C).

**Figure 4:**
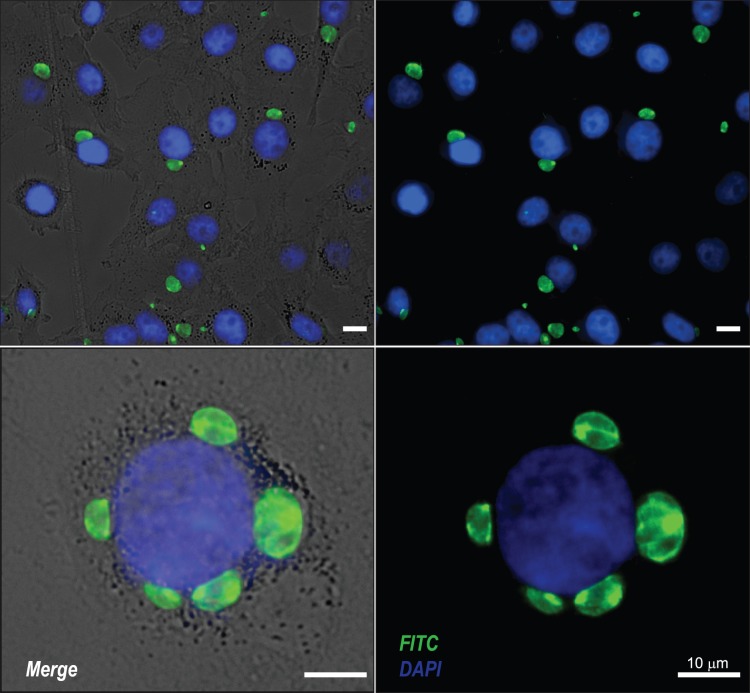
Immunocytochemistry for *T. gondii* using polyclonal IgY anti-STAg. HeLa cells infected with tachyzoites were fixed, permeabilized and incubated with IgY anti-STAg and rabbit IgG anti-IgY conjugated to fluorescein isothiocyanate (FITC). In fluorescence photomicrographs, or merged images with phase contrast. tachyzoites were detected into cell cytoplasm around the nucleus (blue, DAPI) with strong staining (arrows).

In cell culture experiments, the antibodies were used in immunocytochemical assays, where monolayers of HeLa cells infected with *T. gondii* were incubated with IgY anti-STAg. In these experiments, we observed isolated or grouped intracellular tachyzoites closely to cell nucleus, detected by an intense fluorescence on the whole tachyzoite surface (Fig. 4). Overlay of fluorescence emitted by the stained parasites and cell nucleus with phase contrast images of the fibroblast monolayer provided a valuable measure of the parasitism of the infected monolayers.

**Figure 5:**
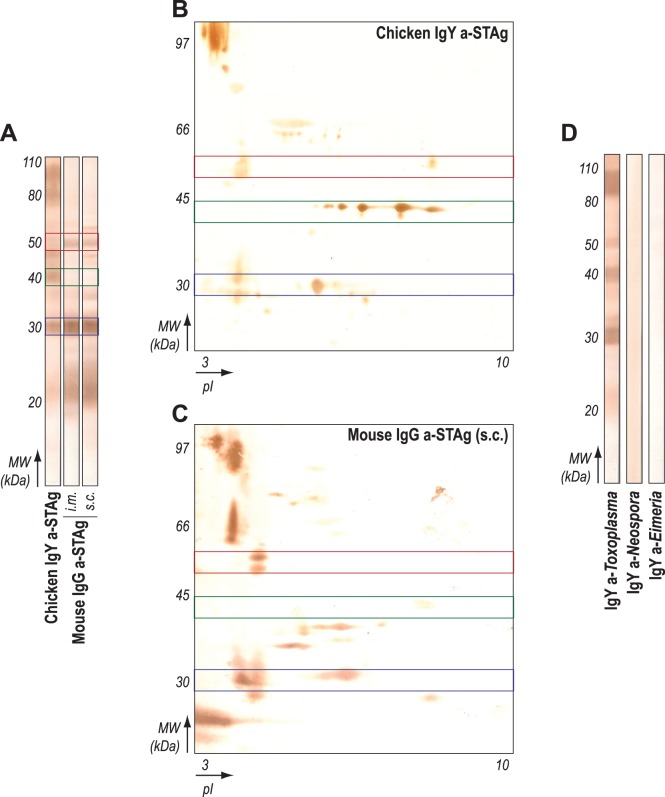
Recognition profile of STAg proteins using polyclonal antibodies raised in chicken and mice. (**A**) Differential reactivity to *Toxoplasma gondii* soluble antigens (STAg) of egg IgY antibodies and mouse serum IgG from animals immunized by intramuscular (i.m.) and subcutaneous (s.c.) routes initially was assessed by 1D immunoblotts (WB1D). Antigens with approximately (∼) 30 kDa (blue box) were evenly recognized by all antibodies tested, which was not observed in antigens with ∼40 kDa (green box) and ∼50 kDa (red box), which were mainly recognized by chicken and mouse antibodies, respectively. The same recognition pattern against STAg proteins was noted in two-dimensional immunoblotts (WB2D), probed with chicken IgY (**B**) or mouse IgG (**C**). (**D**) IgY antibodies obtained from eggs of chickens immunized with *T. gondii*, as well as IgY against *Neospora caninum* and *Eimeria* spp., were assayed against STAg-immobilized nitrocellulose membranes in order to check possible antibody cross-reactivity.

We next observed the repertoire of the polyclonal antibodies raised in avian and mammalian models. Although p30 was evenly recognized by antibodies from the distinct species by one-dimensional immunoblotts (WB1D), differential protein recognition pattern was noted (Fig. 5A). Only chicken-derived antibodies recognized proteins with approximate molecular weights of 40 kDa, while IgG anti-STAg from mice reacted strongly to ∼50 kDa antigen. These differences were maintained independently of the route used to immunize the mice (i.m. or s.c., Fig. 5A). In order to further observe those differences in recognition, we performed 2D-immunoblots (WB2D) using the polyclonal antibodies obtained from immunized hens and mice. The differences in recognition were clearly noted in WB2D assay, with IgY antibodies recognizing a sequence of five antigenic spots at ∼40 kDa, however with distinct isoelectric points – from neutral to basic pH (Fig. 5B). On the other hand, mouse IgG reacted strongly to two acidic antigens at ∼50 kDa (Fig. 5C), while IgY reacted faintly to the same spots and a basic protein with similar molecular weight (Fig. 5B). Such differences were also noted in the distinct recognition patterns in antigens with 60–80 kDa, as well as the absence of immunodominant p22 antigen by chicken IgY. Additionally, STAg immobilized in nitrocellulose strips were probed to anti-*Neospora caninum* e anti-*Eimeria* spp. IgY in order to check the specificity of the antibodies. The assay revealed that IgY antibodies against the closely related protozoa did not cross-react with *Toxoplasma* antigens (Figure 5D).

## Discussion

The use of specific antibodies produced in mammals against antigenic targets and cell markers are essential tools for the study of host-parasite relationships. Unfortunately, these antibodies may be painstaking to obtain, and depend on animal bleeding in order to retrieve the desired immunoglobulins [18]. Chickens present antibody production kinetics and avidity maturation similar to mammals, and IgY antibodies appear in serum approximately 4 to 7 days after inoculation of antigen [9].

In the present study, we produced and purified polyclonal anti-*T. gondii* antibodies from STAg-immunized hens. These anti-STAg antibodies were obtained from the egg yolk, in a protocol which animal bleeding turned out to be unnecessary [19]. Generally, yolk IgY arise around 7^th^ to 10^th^ day after the sera antibodies, in a sera concentration-dependent manner [20]. In addition, the large amounts of polyclonal antibodies recovered from the egg yolk in this process, the high avidity of the produced IgY antibodies, and their potential applications are itself the great features of the protocol herein described. Egg yolks contain large amounts of IgY passively transferred by hens, with mass ranging between 2–8 mg per mL after purification, and it has been estimated that 2–10% of total egg yolk antibodies are antigen-specific [21], [9]. To obtain STAg-specific IgY we used simple protocols, which are easily reproduced without the need of refined equipment or reagents. Additionally, installations required for IgY production are smaller and easier to handle than those designed for small ruminants, commonly used for large scale antisera production [22]. The production of specific IgY has been previously described for the recognition of a broad range of targets, including *Plasmodium falciparum* [23], hepatitis A virus [24] and tumor cells [25]–[26].

Avidity maturation of an antibody represents an increasing affinity interaction between antibodies and antigens. However, this maturation is slowly induced in mammals [27]. We demonstrated that STAg-immunized hens produced large amounts of high avidity polyclonal IgY anti-STAg antibodies after the first booster, with early recognition of p30 proteins. It is known that SAG1 protein (p30) triggers the human IgG maturation in latter stages of *T. gondii* infection [28]. Additionally, IgY antibodies recognized a broad range of proteins, with distinct isoelectric points determined by 2D-immunoblot. Previous studies have shown that serum samples from naturally infected humans react to a broad spectrum of *T. gondii* proteins [29] and experimentally infected mice produce specific antibodies against high and low molecular weight antigens of tachyzoites [30]. Consistently, our results demonstrated that IgY anti-STAg presented an antigen recognition profile similar to mouse serum, although some antigens were more immunogenic for hens than mice, probably due to the phylogenetic distance among these animals. In addition, IgY antibodies raised against closely related parasites did not recognize STAg proteins, which denotes an interesting feature of the IgY system, since cross-reactivity is a normal feature of mammalian IgG.

Hassl and colleagues (1987) [31], in a preliminary study, demonstrated some of characteristics of anti-*T. gondii* IgY and also compared protocols for antibody production and purification, however it was not demonstrated the possible applications of these antibodies to study the parasite. We herein demonstrated for the first time distinct applications for specific IgY antibodies in the *T. gondii* model. As previously stated, this antibody producing system offers high yield of specific antibodies [32]. We believe that immunization of hens with selected *T. gondii* targets could improve the range of applications for IgY antibodies. For the *P. falciparum* model, it has been demonstrated that monospecific and polyclonal IgY antibodies were advantageous [10]. In that sense, immunizations using single antigens of *T. gondii* could be employed to better understand the role of the selected molecules, as tools to improve the diagnosis of acute toxoplasmosis [33] or the knowledge of host-parasite interaction mechanisms [34]. Additionally, the identification of conserved surface antigens among apicomplexan organisms is usually performed by mammal specific IgG antibodies and contributes to our understanding of parasite evolution [35]–[37].

As chickens are phylogenetically distant from mammals and have been shown to be refractory to *T. gondii* infection [38], and differential recognition profile by antibodies from both classes of animals has already been described [39]. The comparative analysis of antigen recognition may be a useful tool to determine different virulence factors of the parasite, thorough proteomic studies for vaccine or diagnostic development. Moreover, the role of *T. gondii* antigens involved in parasite virulence has been largely addressed over the last decade [40]–[41]. The most common protocols used for that purpose are the incubation of the parasite with specific antisera or the generation of strains with targeted depletions, preventing molecular interactions between host and parasite proteins [16].

As shown in this study, IgY antibodies may also be used in this context and further studies are necessary to determine whether these specific antibodies could be employed in therapeutical protocols during acute toxoplasmosis, especially in immunizations performed with single antigens. In conclusion, our results demonstrated that polyclonal IgY anti-STAg antibodies are a promising complementary tool for studies of the *T. gondii* infection model.

## Materials and Methods

### Hens and Mice

Laying hens of the Isa Brown lineage, 25 weeks of age, were used for immunization protocols. Hens were kept in individual cages and received commercial rations and water *ad libitum*. C57BL/6 mice, 6–8 weeks old, were infected with *T. gondii* ME-49 strain to obtain parasite positive brain tissue sections [42] and immunized with STAg to obtain polyclonal IgG antibodies anti-*T. gondii* [43]. All animal procedures were approved by the institutional ethics committee in animal experimentation (Comissão de Ética no Uso de Animais da Universidade Federal de Uberlândia - Protocol No. 107/11), and were performed based on the Ethical Principles in Animal Research adopted by the Brazilian College of Animal Experimentation and instructions disposed in the 2000 Report of the AVMA Panel on Euthanasia [44].

### Soluble Tachyzoite Antigen Preparation

Soluble tachyzoite antigen (STAg) was obtained using a previously described protocol [45]. Briefly, tachyzoites of *T. gondii* (RH strain) were maintained by serial passage in BALB/c mice and obtained from peritoneal exudates from mice infected 48 h earlier. Only exudates containing 100% of free-ranging tachyzoites were used for antigen preparation. Parasite suspension was washed in phosphate buffered saline (PBS, pH 7.2), added to a protease inhibitor cocktail (Complete Ultra tablets, Roche, USA), processed through lysis by repeated freezing and thawing cycles, sonicated, and centrifuged at 14,000×*g* for 30 min at 4°C. After supernatant recovery, total protein was estimated by the Bicinchoninic acid kit (BCA, Sigma, St. Louis, MO, USA) and aliquots were stored at −80°C until use. The same protocol was performed with cells extracted from the peritoneal cavity of mice, used as experimental controls.

### Immunization Protocols

Hens (n = 4) were immunized by muscular route with emulsion composed of STAg and Freund’s adjuvant, according to previously described protocol [46]. Primary immunization was performed with 100 µg of STAg in 250 µL of PBS and equal volume of Freund’s complete adjuvant (Sigma). Two boosters were performed at 15 day intervals, with 100 µg of STAg plus Freund’s incomplete adjuvant. In parallel, *Neospora caninum* (n = 4) and PBS (n = 2) -immunized hens were maintained for parasite-specific and irrelevant IgY purification, respectively. Hens were monitored daily for adverse effects and individual laid eggs were daily collected and stored at 4°C until further processing.

In addition, C57BL/6 mice were immunized by intramuscular and subcutaneous routes with STAg, for comparative purposes. Primary immunization was performed with 25 µg of STAg in 50 µL of PBS and equal volume of Freund’s complete adjuvant (Sigma). Two boosters were performed at 15 day intervals, with 25 µg of STAg with Freund’s incomplete adjuvant. Serum samples were collected weekly and stored at −20°C until use.

### Purification of Egg Yolk Antibodies

IgY was purified by the water-dilution method as previously described [19]. In order to obtain a representative sampling of antibody production along the weeks after immunization, eggs laid weekly from each hen were pooled prior to IgY extraction. Then, egg white was removed and egg yolk was diluted 10-fold in deionized ultrapure water adjusted to pH 5.0–5.2 with 1N HCl and homogenized thoroughly. After centrifugation at 10.000×g for 25 min at 4°C, the supernatant was collected, consisting of lipid-free fraction (S1). S1 was then precipitated with the addition of 19% sodium sulphate (w/v). After centrifugation (10.000×g, 25 min, 4°C), the pellet was retrieved and represented the IgY-enriched fraction (P2). P2 samples were resuspended and dialyzed against PBS to eliminate residual salt. Additionally, P2 samples were submitted to exclusion-size gel chromatography using Sephacryl S-300 column (GE Healthcare, Uppsala, Sweden), at a flow rate of 3 mL/min, and the IgY-enriched protein fraction was determined by 280 nm readings. Actual protein concentration was measured by BCA kit (Sigma) and samples were stored at −20°C until use. This protocol was also applied to extract IgY antibodies from the eggs of hens immunized *in house* with *N. caninum* soluble antigen, as well as from commercially available egg yolk powder containing anti-*Eimeria spp.*(*E. acervulia, E. maxima e E. tenella*) IgY antibodies (Supracox, Investigación Aplicada, Sociedad Anónima de Capital Variable, Puebla, Mexico). Possible cross-reactivity of the purified IgY antibodies to mouse proteins were ruled out by immunoblots (Fig. S2).

The quality of the purification protocols was analyzed in polyacrylamide gel electrophoresis with sodium dodecyl sulphate (SDS-PAGE) at 8% in non-reducing conditions. IgY enrichment was assessed by slot dot assays, as follows: proteins samples obtained during IgY extraction were sequentially diluted 10-fold (10 µg to 10^−4^ µg) and transferred to nitrocellulose membrane by a vacuum apparatus (Bio-Dot SF; Bio-Rad, EUA). The presence of detectable chicken antibodies was verified by an rabbit anti-IgY antibody conjugated to peroxidase (Sigma) as secondary antibody, revealed by H_2_O_2_ and DAB (Sigma).

### Indirect ELISA for Detection of Antibodies Anti-STAg

The kinetics of chicken IgY anti-STAg production and avidity maturation were evaluated by an indirect ELISA. The optimal conditions for ELISA were obtained through block titration of the reagents. Briefly, high affinity microtiter plates (Costar Corning Incorporated, USA) were coated with STAg (10 µg/mL) in 0.06 M carbonate buffer (pH 9.6) and incubated overnight at 4°C. Plates were washed 3 times with PBS-Tween 0.05% (PBS-T) and blocked with PBS-T plus 1% bovine serum albumin (PBS-T-BSA) for 1 h at room temperature. P2 samples were adjusted to 2 µg/well in PBS-T- BSA, added to the wells in duplicate and incubated for 1 h at 37°C. After washing, plates were incubated with rabbit anti-IgY antibody labeled with peroxidase, diluted 1∶30.000 in PBS-T, for 1 h at 37°C. The reaction was revealed by adding 0.01 M 2,2′-azino-bis(3-ethylbenzthiazoline-6-sulphonic acid) (ABTS, Sigma) and 0.03% H_2_O_2_, and optical density (OD) was determined at 405 nm. Two positive quality-controls and six negative controls (irrelevant IgY) were included in each plate in order to calculate the cut off, which was established as the mean OD values for irrelevant IgY plus three standard deviations. The same protocol was applied to mouse IgG anti-STAg obtained after immunization, using a goat IgG anti-mouse IgG conjugated to peroxidase (Sigma), at 1∶2000 dilution.

Results were expressed as ELISA index (EI) as previously proposed [47] as follows: EI = OD_sample_/OD_cut off_, where values of EI >1.2 were considered positive.

To measure avidity maturation, plates were coated and blocked as described above. After the addition of P2 samples in quadruplicates, two of the wells were rinsed with 6 M urea in PBS-T, whereas the other duplicate wells were rinsed with PBS-T only for 10 min at room temperature. Next, all wells were washed 3 times in PBS-T and subsequent steps were performed as described for the regular indirect ELISA.

### 1D- and 2D-immunoblot Assays

To investigate the antigen recognition repertoire of IgY anti-STAg antibodies, immunoblot assays were carried out along with avidity maturation analysis against STAg. In 1D-immunoblot, STAg was separated on 12% SDS-PAGE under non-reducing conditions, and electrotransferred to nitrocellulose membranes. Non-specific interactions were blocked by 5% skim milk-PBS-T incubation, for 2 h at room temperature. Nitrocellulose strips were then incubated with P2 samples from weekly egg yolk pools adjusted to 2 µg of IgY. In parallel, avidity maturation was measured by strip treatment with 6 M urea for 10 min at room temperature. IgY was detected by incubating the secondary antibody, rabbit anti-IgY labelled with peroxidase, diluted at 1∶20.000, for 2 h at room temperature. Reaction was revealed by adding 10 mg of 3,3′- diaminobenzidine tetrahydrochloride (DAB, Sigma) in 15 mL of Tris buffered saline (TBS) and 12 µL of 30% hydrogen peroxide (Sigma). Reaction was stopped with distilled water. Experiments with similar conditions were performed for IgY anti-N. caninum and anti-Eimeria spp., as well as for the establishment of recognition kinetics of mouse IgG obtained from mice immunized by i.m. and s.c. routes (1∶100 sera dilution; 1∶2000 anti-mouse IgG conjugated to peroxidase – Sigma).

A 2D-immunoblot assay was also carried out to evaluate the antigen recognition profile of immunized hens to STAg. Briefly, 60 µg of STAg dialysed in ultrapure water, was separated by isoelectric focusing (IEF) on 7-cm immobilized pH gradient strips (ReadyStrip™ IPG Strip pH 3–10) overnight at room temperature, following the manufacturer instructions for equipment and reagents (GE, Healthcare, Uppsala, Sweden). After IEF, strips were equilibrated and loaded onto precast 12% polyacrylamide gels. Electrophoresis was performed and 2D-gels were stained with Coomassie brilliant blue G-250® (Sigma) or electrotransferred to nitrocellulose membranes. 2D-immunoblot was performed as described above for 1D-immunoblot.

In parallel, to compare the STAg recognition profile between mammalian and avian hosts, immobilized STAg membranes were probed with sera of experimentally *T. gondii* immunized mice, diluted 1∶100 in 1% skim milk in PBS-T. Primary antibodies were detected by goat anti-mouse IgG antibody labeled with peroxidase (1∶1000, Sigma) for 2 h at room temperature. Reaction was revealed with DAB and stopped with distilled water.

### Immunocytochemistry and Immunohistochemistry

In order to detect *T. gondii* forms on paraffin-embedded brain tissue sections of mice chronically infected with *T. gondii* ME-49 strain [42], we carried out an immunohistochemical assay by using IgY anti-STAg as primary antibodies. Brain sections were deparaffinized and incubated with specific IgY (30 µg/mL) diluted in PBS for 30 min at 37°C. After washing, slides were incubated with rabbit anti-IgY labeled with fluorescein isothiocyanate (FITC, Sigma) diluted 1∶300 in addition to DAPI for 60 min at 37°C. Slides were mounted with carbonate-buffered glycerin (pH 9.0) and read at an inverted fluorescence microscopy system (FSX-100, Olympus, Tokyo, Japan).

In order to detect intracellular *T. gondii* replication, we standardized a cell culture based immunocytochemical assay using IgY anti-STAg as primary antibodies. Briefly, HeLa cells (ATCC No. CCL-2) cultured in 24-well plates were incubated with tachyzoites of *T. gondii* RH strain in a multiplicity of infection (MOI) equal to 1, in RPMI-1640 medium supplemented with 10% fetal calf serum for 24 h at 37°C and 5% CO_2_ atmosphere. After incubation, cells were fixed with 4% formaldehyde and permeabilized with 0.1% Triton-X 100, followed by the incubation with the specific IgY antibody (30 µg/mL) for 2 h at 37°C. The next step consisted of the incubation with rabbit anti-IgY antibody labelled with FITC (Sigma), diluted 1∶600 in PBS plus 4′,6-diamidino-2-phenylindole (DAPI, Sigma) for 1 h at 37°C. The reaction was read in an inverted fluorescence microscope system (EVOS, AMG Microscopy Group, USA).

### Statistical Analysis

Statistical analysis was performed using the GraphPad Prism version 4.0 software (GraphPad Software Inc., La Jolla, CA, USA). The Student *t* test was used to investigate significant differences between groups. Values of *P*<0.05 were considered statistically significant.

## Supporting Information

Figure S1 **Production kinetics of mouse serum IgG anti-STAg antibodies.** (**A**) Indirect ELISA showing kinetics of mouse IgG recognition of soluble tachyzoite antigens (STAg) during the immunization protocol by intramuscular (i.m.) route. Black arrows indicate the three immunization doses. Samples with ELISA Index (EI) equal or superior to 1.2 were considered positive. (**C**) 1D-immunoblot presenting recognition kinetics (from 0 to 49 days p.i.) of STAg proteins by serum IgG of immunized mice.
(EPS)

Figure S2 **IgY antibodies derived from immunization protocols are not reactive to antigens from mouse peritoneal cells.** Polyclonal IgY antibodies against *Toxoplasma gondii*, *Neospora caninum* and *Eimeria* spp. were assayed against soluble antigens of peritoneal cells to assure the specificity of the protocol described in this work.
(EPS)
